# Hijacker of the Antitumor Immune Response: Autophagy Is Showing Its Worst Facet

**DOI:** 10.3389/fonc.2016.00246

**Published:** 2016-11-18

**Authors:** Elodie Viry, Muhammad Zaeem Noman, Tsolère Arakelian, Audrey Lequeux, Salem Chouaib, Guy Berchem, Etienne Moussay, Jérôme Paggetti, Bassam Janji

**Affiliations:** ^1^Laboratory of Experimental Cancer Research, Department of Oncology, Luxembourg Institute of Health, Luxembourg City, Luxembourg; ^2^INSERM U1186, Gustave Roussy Cancer Center, Villejuif, France; ^3^Department of Hemato-Oncology, Centre Hospitalier du Luxembourg, Luxembourg City, Luxembourg

**Keywords:** autophagy, hypoxia, antitumor immune response, tumor microenvironment, Natural Killer cells, Cytotoxic T Lymphocytes, chloroquine and immunological checkpoint-based immunotherapy

## Abstract

Macroautophagy (hereafter referred to as autophagy) is a housekeeping process constitutively executed at basal level in all cells to promote cellular homeostasis by regulating organelle and protein turnover. However, autophagy deregulation caused by several stress factors, such as hypoxia, is prevalent in many cancers. It is now well established that autophagy can act as tumor suppressor or tumor promoter depending on tumor type, stage, and genetic context. In developed tumors, autophagy promotes the survival of cancer cells and therefore operates as a cell resistance mechanism. Emerging evidence point to the prominent role of autophagy in disabling the antitumor immune response by multiple overlapping mechanisms leading to tumor escape from immune cell attack mediated by both natural killer cells and cytotoxic T-lymphocytes. Such a role has inspired significant interest in applying anti-autophagy therapies as an entirely new approach to overcome tumor escape from immune surveillance, which constitutes so far a major challenge in developing more effective cancer immunotherapies. In this review, we will summarize recent reports describing how tumor cells, by activating autophagy, manage to hijack the immune system. In particular, we will focus on the emerging role of hypoxia-induced autophagy in shaping the antitumor immune response and in allowing tumor cells to outmaneuver an effective immune response and escape immunosurveillance. In keeping with this, we strongly believe that autophagy represents an attractive future therapeutic target to develop innovative and effective cancer immunotherapeutic approaches.

## Introduction

### Autophagy and Hypoxia

Under physiological conditions, autophagy is executed at low level to degrade damaged proteins and/or organelles in order to sustain metabolism and cell homeostasis. The level of basal autophagy varies depending on the tissue type and some tissues are particularly dependent on autophagy (e.g., brain, liver, and muscle) (1). It is now generally appreciated that autophagy is deregulated in some pathological conditions including cancer and it seems that the role of autophagy in cancer is context and circumstance-dependent (2). Thus, autophagy suppresses tumor progression by limiting chromosomal instability (3); however, established tumors appear to utilize autophagy in order to survive periods of metabolic or hypoxic stress (3). In line with its role in sustaining viability and conferring stress tolerance, targeting autophagy has inspired significant interest for mitigating tumor growth and/or restoring response to anticancer therapies. Consistent with the part of autophagy in promoting tumor progression, it has been shown that targeting autophagy reduces cell migration and invasion *in vitro* and attenuates metastasis *in vivo* in a breast cancer mouse model by promoting focal adhesion disassembly through targeted degradation of paxillin (4). Furthermore, several new studies have reported that autophagy activation plays also a major role in tumor immunity. Autophagy enhances tumor antigens processing and presentation thereby promoting adaptive antitumor immunity. In antigen-presenting cells (APC), autophagy promotes antigen presentations by major histocompatibility complexes (MHC). Such antigens presented by MHC class II and I are recognized by CD4+ and CD8+ T cells, respectively, in order to induce specific cytotoxic T-lymphocyte (CTL)-mediated immune response. Thus, autophagy allows the traffic of engulfed antigens to endosomes, where they are digested by cathepsins in order to be loaded onto MHC class II molecules and translocate to the plasma membrane to be finally presented to CD4+ T cells (5). Based on the role of autophagy in antigen presentation and processing, it has been proposed that autophagy plays a beneficial role for the induction of antitumor immunity. In this context, hypoxia has been reported to increases tumor cell shedding of MHC class I resulting into increased resistance to natural killer (NK)-mediated lysis (6) *via* increased expression of ADAM10 (7). The role of autophagy in antigen processing and presentation was reported in several comprehensive reviews (8–12) and will not be the subject of this review. However, emerging evidence strongly suggest that autophagy shows its worst facet when induced within the hypoxic tumor microenvironment (13). Indeed, autophagy impairs the antitumor immune responses mediated by CTL and NK cells and has been reported to enhance the immunosuppressive properties of myeloid-derived suppressor cells (MDSCs). These issues will be discussed in more detail in the present review.

It is now well established that hypoxia develops due to a mismatch between tumor growth and neovascularization. Cellular responses to hypoxia are mediated by hypoxia-inducible factor (HIF) family of transcription factors. Both HIF-1 and HIF-2 are composed of two subunits: an O_2_ regulated subunit (HIF1-α and HIF2-α) and a constitutively expressed subunit (HIF1-β and HIF2-β) (14). In the presence of oxygen, HIF-1α is hydroxylated by prolyl hydroxylase domain protein 2 (PHD2) on a proline residue leading to an interaction with the Von Hippel-Lindau (VHL) protein. Therefore, HIF-1α is polyubiquitylated and consequently targeted for degradation by the ubiquitin proteasome system. Under hypoxic conditions, HIF-1α is stabilized, accumulated in the cytoplasm, and then translocated to the nucleus where it can form a heterodimer complex with HIF-1β. Finally, HIF-1 binds to hypoxia response elements (HREs) on the chromatin in order to induce the transcription of more than 300 genes, involved in many biological processes, including angiogenesis, cell survival, metastasis, stem cell-like phenotype, and immune escape (15).

**Figure 1:**
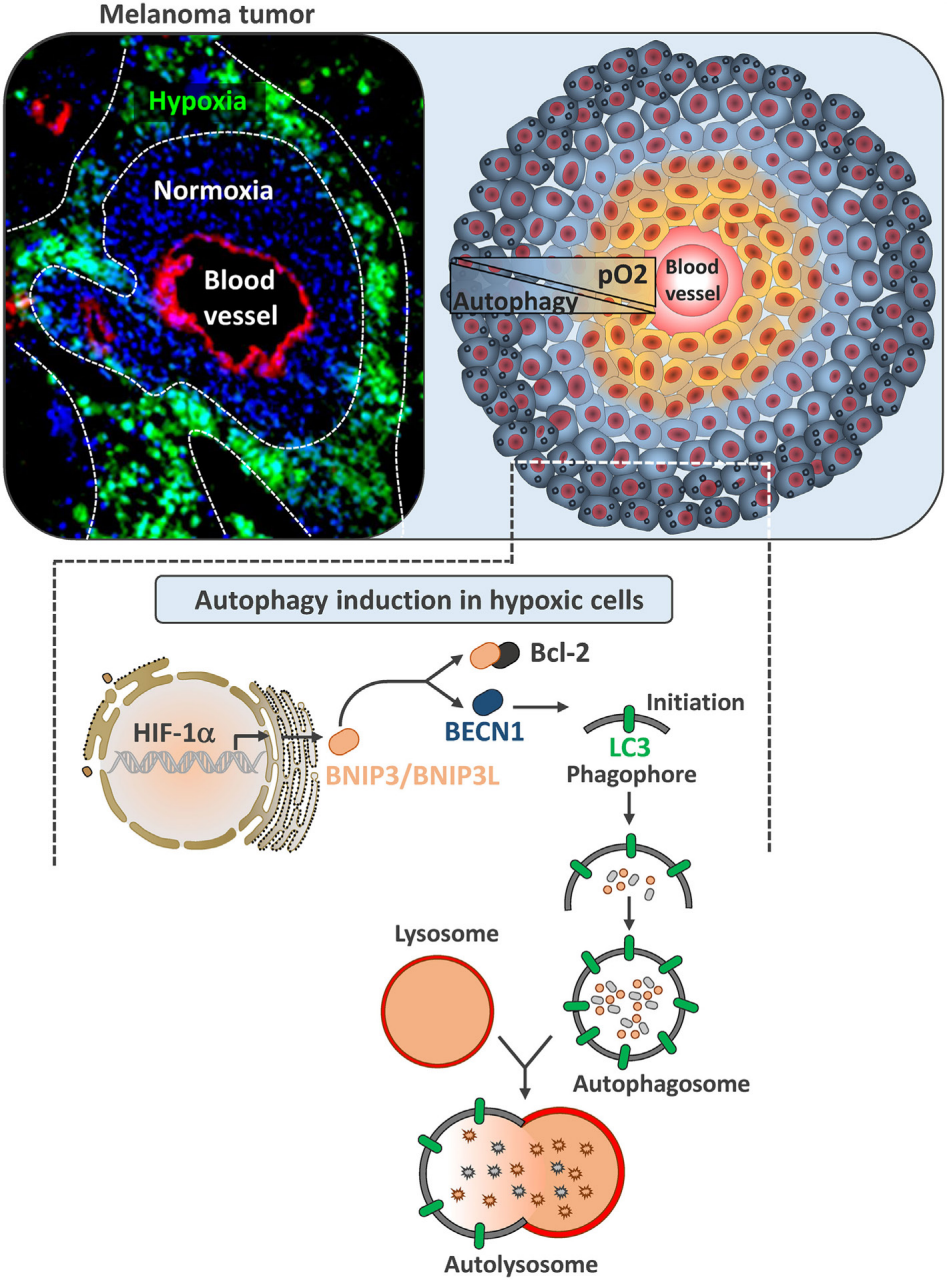
**Autophagy induction in hypoxic melanoma cells**. Left image represents B16-F10 tumor section transplanted in C57BL/6 mouse. Tumor section was stained with Pimonidazole to illustrate hypoxic zones (in green). Blood vessels were stained with anti-CD31 (red) and nuclei with DAPI (blue). Right image is a schematic representation of tumor showing normoxic tumor cells close to the blood vessel (yellow) and hypoxic tumor cells (blue) at the periphery. In B16-F10 mouse melanoma, autophagy is selectively induced in hypoxic zones. Lower image represents the mechanism underlying the activation of autophagy by hypoxia. In hypoxic cells, hypoxia-dependent activation of HIF-1α upregulates BNIP3 and BNIP3L by binding to their promoter regions. Both of these BH3-only proteins (BNIP3 and BNIP3L) disrupt the complex between Beclin1 and Bcl-2, leading to the induction of autophagy.

Three major pathways have been reported to induce autophagy under hypoxia. Briefly, HIF-1α-mediated induction of the expression of the BH3-only protein Bcl-2/adenovirus E1B 19 kDa-interacting protein 3 (BNIP3) and the related protein, BNIP3L (16). BNIP3 and BNIP3L disrupt the autophagy inhibitory complex between Beclin1 (BECN1) and B-cell lymphoma 2 (Bcl-2) and activate autophagy in hypoxic cells, which operate as an adaptive survival response during prolonged hypoxia (Figure 1) (17). Autophagy can also be activated by endoplasmic reticulum (ER) stress in cancer cells. Indeed, ER stress stimulates the assembly of the pre-autophagosomal structures, the formation of autophagosomes, and the transport to the vacuoles in an autophagy-related gene (ATG) dependent manner (18). Autophagy can also be induced in hypoxic cells subjected to metabolic stress through the activated adenosine monophosphate-activated protein kinase (AMPK). This leads to the initiation of autophagy both directly and indirectly by inhibiting the mammalian target of rapamycin (mTOR) (19, 20).

### Immune Checkpoints and Hypoxia

Immune checkpoint-based cancer immunotherapy has now emerged as a promising revolutionary treatment for many cancers including, but not limited to, melanoma, lung cancer, kidney cancer, bladder cancer, prostate cancer, and lymphoma (21). In the near future, immune checkpoint-based cancer immunotherapies will most probably join the ranks of surgery, radiation, chemotherapy, and targeted therapy as a pillar of classical anticancer therapies. Ipilimumab (anti-CTLA-4) was approved in 2011, and pembrolizumab and nivolumab (anti-PD-1) were approved in 2014 by U.S. Food and Drug Administration (FDA) for the treatment of melanoma. These antibodies will soon be approved for treatment of patients with lung cancer, kidney cancer, and many other tumor types. The fact that these antibodies do not target tumor cells, but rather remove inhibitory signals on antitumor T cells have led to durable clinical responses in some patients (21).

**Figure 2:**
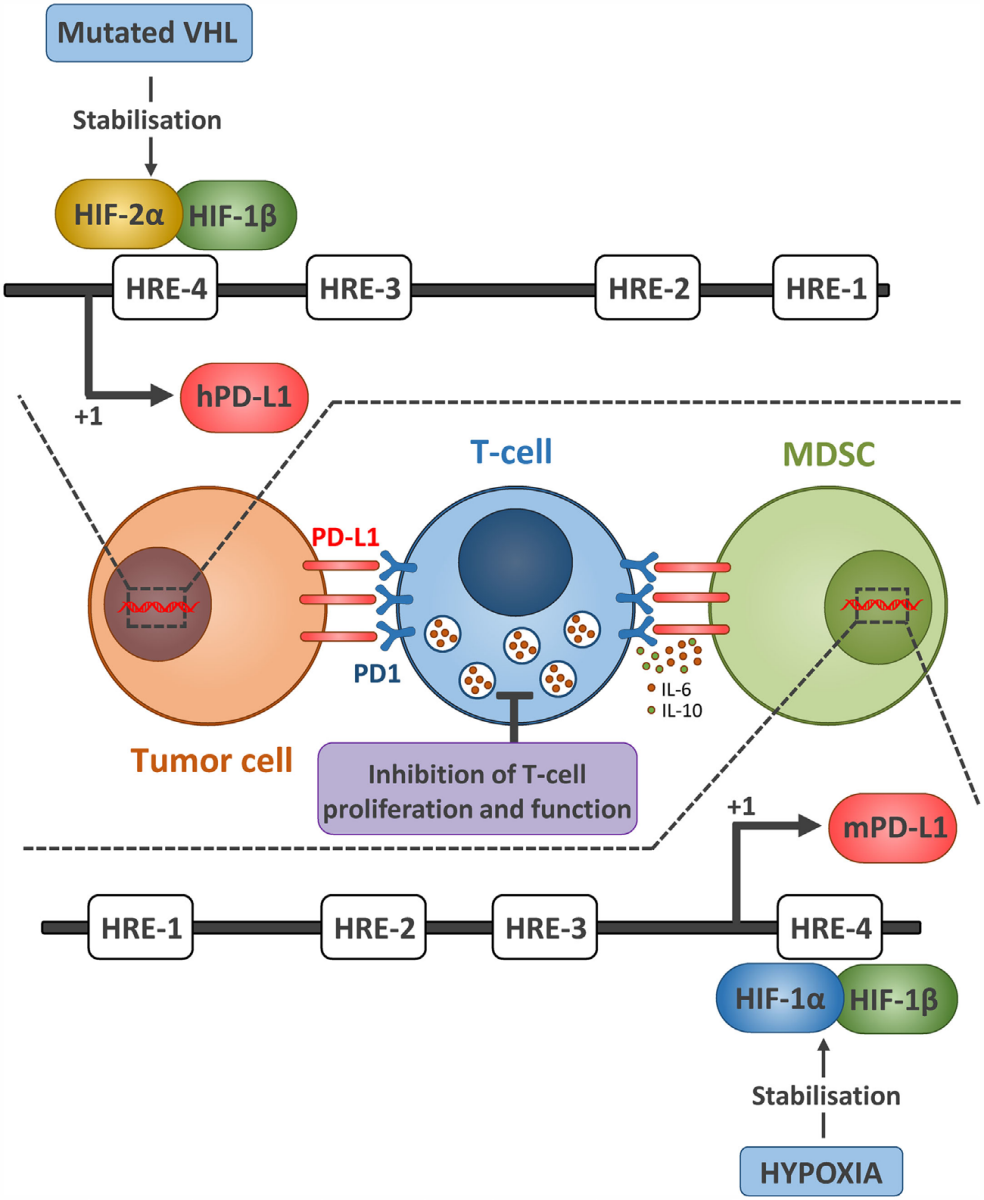
**Hypoxia *via* HIF-1α and HIF-2α regulates the expression of PD-L1 expression on tumor cell and MDSCs**. The upper part represents the mechanism by which HIF-2α regulates the expression of PD-L1 in ccRCC tumor cells. Due to mutated VHL in ccRCC tumors, HIF-2α is constitutively stabilized and activated. HIF-2α translocates to the nucleus, binds to the HRE-4 in human PD-L1 promoter, and upregulates its expression. Whether this PD-L1 confers resistance to ccRCC sensitivity to antitumor effector cells remains to be investigated. The lower part represents the mechanism by which hypoxia *via* HIF-1α regulates the expression of PD-L1 in MDSCs. Similarly, stabilized HIF-1α in MDSCs isolated from tumors bound directly to the HRE-4 in the PD-L1 proximal promoter in MDSCs. The immune suppressive function of MDSCs, enhanced under hypoxia, was abrogated following PD-L1 blockade and hypoxia-mediated upregulation of IL-6 and IL-10 in MDSCs was significantly attenuated after PD-L1 blockade.

Programed death 1 (PD-1) is an inhibitory receptor expressed mostly on activated T cells as well as other immune cells, and its expression is associated with T cell exhaustion. PD-1 has two ligands, Programed death-ligand 1 and 2 (PD-L1 and PD-L2). The interaction between PD-1 and its ligands sends a negative signal to T cells to dampen the antitumor immune response. Antibody-based blockade of PD-1 was shown to enhance effector T cell responses and induce T cell–mediated tumor rejection in some mouse models (22). PD-1 or PD-L1 blockade has been proven to be successful in many cancers (21), and anti-PD-1 antibodies have recently been approved for use in the United States and Asia. Hypoxia-inducible factors were shown to enhance the effector responses (including both costimulatory and inhibitory molecules) of VHL deficient CD8+ T cells (with constitutive HIFs) to persistent viral antigen (23). We and others have reported that HIF-1α (24) and HIF-2α (25) regulate PD-L1 expression under hypoxia (Figure 2). Hypoxia through HIF-1α has been also shown to regulate functional CD137 (4-1BB) on tumor-infiltrating T lymphocyte (26) and more recently soluble CD137 in malignant tumor cells (27).

Indeed, we first found a differentially higher expression of PD-L1 on tumor-infiltrating MDSCs as compared to splenic MDSCs (24) and hypoxia dramatically and significantly increased the percentage of PD-L1 positive MDSCs isolated from spleen in different tumor-bearing mice. We further provided evidence that HIF-1α is a major regulator of PD-L1 mRNA and protein expression, and that HIF-1α regulates the expression of PD-L1 by binding directly to the HRE-4 in the PD-L1 proximal promoter. The immune suppressive function of MDSCs, enhanced under hypoxia, was abrogated following PD-L1 blockade and hypoxia-induced upregulation of interleukin-6 and 10 (IL-6 and IL-10) in MDSCs was significantly attenuated after PD-L1 block (24).

More recently, we showed that tumors from clear cell renal cell carcinoma (ccRCC) patients displaying VHL biallelic inactivation (i.e., loss of function) exhibit a significant increase in PD-L1 expression as compared to ccRCC tumors carrying one VHL wild-type allele. Using the inducible VHL 786-O-derived cell lines with varying HIF-2α stabilization levels, we showed that PD-L1 expression levels positively correlate with VHL mutation and HIF-2α expression. Targeting HIF-2α decreased PD-L1 while HIF-2α overexpression increased PD-L1 mRNA and protein levels in ccRCC cells. Interestingly, chromatin immunoprecipitation and luciferase assays revealed a direct binding of HIF-2α to a transcriptionally active HRE in the human PD-L1 proximal promoter in 786-O cells. In conclusion, VHL mutations positively correlate with PD-L1 expression in ccRCC and may influence the response to ccRCC patients to anti-PD-L1/PD-1 immunotherapy (25).

## Effect of Autophagy on Antitumor Immune Responses

The role of cancer cell-associated autophagy in the modulation of the antitumor immune response takes place at different levels. First, autophagy may act at early step of cancer development, thus regulating the immune surveillance in the context of tumorigenesis. Second, at later stage of cancer, the autophagy process was identified as an important modulator of the tumor cell proteome and secretome, allowing cancer cells to communicate with neighboring cells in the tumor microenvironment. Finally, cancer cell-associated autophagy may also acts as an intrinsic resistance mechanism evolved by tumor cells to overcome immune cell attack. Mounting evidence in the literature suggests that depending on the context, the cancer model, and the type of stressor (e.g., hypoxia, chemotherapy), autophagy might either assist or prevent anticancer immunity.

### Autophagy Modulates the Immune Surveillance during Tumorigenesis

Autophagy serves as an adaptive response during period of stress by maintaining cellular integrity and metabolic homeostasis. Extensive works coming from Eileen White’s group have demonstrated the crucial role of autophagy in cancer tumorigenesis. Notably, autophagy process actively contributes to elimination of source of genotoxic stress in order to maintain genomic integrity (28–30). However, autophagy inhibition may halt tumorigenesis not only by affecting cancer cell metabolism and proliferation *per se* but also by enhancing the antitumor immunosurveillance. The recent study of Rao et al. illustrated the complex role of autophagy in either tumorigenesis or tumor progression depending on the stage of the disease (31). Inactivation of Atg5-dependent autophagy increased the number of tumor foci and favored the progression from hyperplasia to adenoma in a murine model of lung cancer (with KrasG12D mutation). However, in later stage of the disease, disabled autophagy reduced the progression from adenoma to adenocarcinoma. Immunoprofiling of early pulmonary lesions indicated that autophagy inactivation did not alter the global infiltration of the CD3ε^+^ T cells, but the amount of infiltrating Foxp3^+^ regulatory T cells (Tregs), which was increased in autophagy-deficient tumor. The authors clearly demonstrated that inactivation of autophagy in pneumocytes carrying a Kras mutation triggered a local expansion of Tregs cells, which control lung tumor initiation. This study established a cause-effect relationship between defective autophagy and infiltration of immunosuppressive Tregs cells. Other studies stated that autophagy inhibition may hamper tumorigenesis also by enhancing antitumor immunosurveillance. Recently, Levy et al. showed that conditional inactivation of the autophagy gene Atg7 in intestinal epithelial cells (IECs) suppresses the development of precancerous lesions in Apc mutant mice (32). The authors demonstrated that CD8+ T cells were essential effectors of the antitumor immune response mediated by the inhibition of autophagy. Autophagy inactivation in IECs favored the infiltration and the expansion of interferon (IFN)-producing CD8+ T cells in the intestinal mucosa and participated in their priming. Moreover, Atg7 deficiency endorsed a CD4+ Th1 signature, which is associated with a good outcome in patients with colorectal cancer (33). However, Tregs infiltration was also induced, but in contrast to most studies, Tregs did not show immunosuppressive properties in those experimental conditions. Interestingly, this study reported that autophagy deficiency in IECs influenced intestinal microbiota, which is a prerequisite for an efficient anticancer immune response (32). In addition, Wei et al. have reported that conditional deletion of the ULK1-associated protein FIP200 in a PyMT-driven breast cancer murine model reduced the initiation of mammary intraepithelial neoplastic lesions and altered tumor progression by enhancing antitumor immune surveillance. FIP200-deficient tumor cells showed an upregulated expression of genes implicated in IFN signaling and an increased production of the chemokine CXCL10 that may favor infiltration of CD8+ T lymphocytes. This study, once again, showed that CD8+ T cells are crucial effectors of the autophagy-induced immunomodulation, as depletion of CD8+ T cells with selective antibodies restored mammary tumor initiation and progression (34, 35).

### The Dual Immunomodulatory Role of Autophagy Induction in Cancer Cells

#### Autophagy Influences the Adaptive Antitumor Immunity

The importance of autophagy was also highlighted during tumor progression to late stage of the disease. Autophagy acts as a pro-survival mechanism allowing cancer cells to overcome stress conditions. However, cells that fail to adapt stress will use autophagy to release specific signaling molecules allowing the immune clearance of damaged cells. Thus, the concept of immunologic cell death (ICD) has rapidly emerged as an important feature determining an effective antitumor immune response (36). ICD is characterized by the rapid surface exposure of calreticulin (ecto-CRT), the secretion of ATP, and the release of apoptotic proteins as high mobility group box 1 (HMGB1), which are excellent immunogenic signals (36, 37). *Bona fide* ICD leads to increased maturation and stimulation of dendritic cells (DCs), which are required for robust T cells response. An increasing amount of evidence suggests that autophagy plays a crucial role during ICD by modulating the cancer cell secretome and surface proteome following stress or cell death induction.

In response to chemotherapeutic stress, autophagy-deficient cells displayed ecto-CRT, released HMGB1 but secreted less ATP than autophagy-competent cells. Michaud et al. showed that autophagy contributes to immunogenic signaling *in vivo* through the secretion of ATP. Impairment of autophagy in mitoxantrone (MTX)-treated murine colorectal carcinoma cells decreased the intratumoral recruitment of DCs and T cells when injected in immunocompetent mice. Artificial increase of intratumoral ATP restored the immunogenicity of MTX-treated autophagy-deficient cells, confirming that activation of autophagy following chemotherapy-induced ICD facilitates ATP secretion and the intratumoral accumulation of DCs and T lymphocytes (38).

Another important immunostimulatory signal that requires autophagy is the exposure of CRT at the cancer cell surface. Ecto-CRT triggers the engulfment of the damaged cells by macrophages. It has been described by Garg et al. that exposure of CRT is abolished in cancer cells when chaperone-mediated autophagy is impaired (39). In contrast to expectations, the same authors demonstrated that ER stress-induced autophagy, following photodynamic therapy, negatively regulated CRT surface exposure without affecting ATP secretion. Inhibition of ER stress-induced autophagy, by knocking down Atg5 in cancer cells, improved the maturation of IL-6 secreting DCs, and thus triggered proliferation of IFNγ-producing CD4+ and CD8+ T cells. Moreover, inactivation of autophagy in untreated melanoma cells increased CRT surface exposure, suggesting that basal autophagy in cancer cells may support immune escape (39, 40).

The pro-inflammatory role of autophagy may also be related to the protein HMGB1. In one hand, the cytosolic form of HMGB1 is able to bind to BECN1, which further leads to the formation of autophagosomes. On the other hand, secreted HMGB1 can also trigger autophagy through the binding to the RAGE receptor (receptor for advanced glycation endproducts) (41, 42). Once released outside the cell, HMGB1 has been shown to own both immunosuppressive and immunostimulatory properties (43). Production of HMGB1 by colon cancer cells also decreases the differentiation of DCs. In line with these experimental results, high HMGB1 levels in primary tumor tissues correlate with low intratumoral CD205+ DCs (44). Conversely, tumor cell-derived HMGB1 was shown to suppress CD8+ T cells antitumor immunity through the induction of IL-10-producing Tregs (45). Recently, Ladoire et al. provided evidence that cytoplasmic microtubule-associated protein 1A/1B-light chain 3 (LC3II), and nuclear HMGB1 expression may influence the nature of the immune infiltrate in breast cancer. Thus, the absence of LC3II correlated with intratumoral, but not peritumoral, infiltration of Foxp3+ Tregs, CD68+ tumor-associated macrophages and less CD8+ T cytotoxic lymphocytes. Moreover, absence of HMGB1 expression in the nuclei was associated with the increase in both intratumoral and peritumoral infiltration by Foxp3+ and CD68+ cells. Taken together, these results suggest that autophagy blockade or HMGB1 loss in breast cancer cells have a negative impact on the anticancer immune surveillance (46).

Autophagy has also emerged as a critical pathway for tumor antigen cross-presentation, which is determinant for the initiation of an efficient adaptive immune response (47, 48). Autophagy actively participates in antigen sequestration and delivery to DCs for cross-priming of CD8+ T cells. Inhibition of autophagy in melanoma cells by knocking down BECN1 significantly reduced T cell proliferation *in vitro* and *in vivo* (47). Moreover, induction of autophagy by the vitamin E derivative α-tocopheryloxyacetic acid (α-TEA) in 3LL Lewis lung carcinoma and 4T1 mammary carcinoma cells led to the release of antigen-containing autophagosomes that increase CD8+ T cells activation (49). Interestingly, tumor antigens packaged into autophagosomes were more efficient for T cells activation than soluble antigens (47).

**Figure 3:**
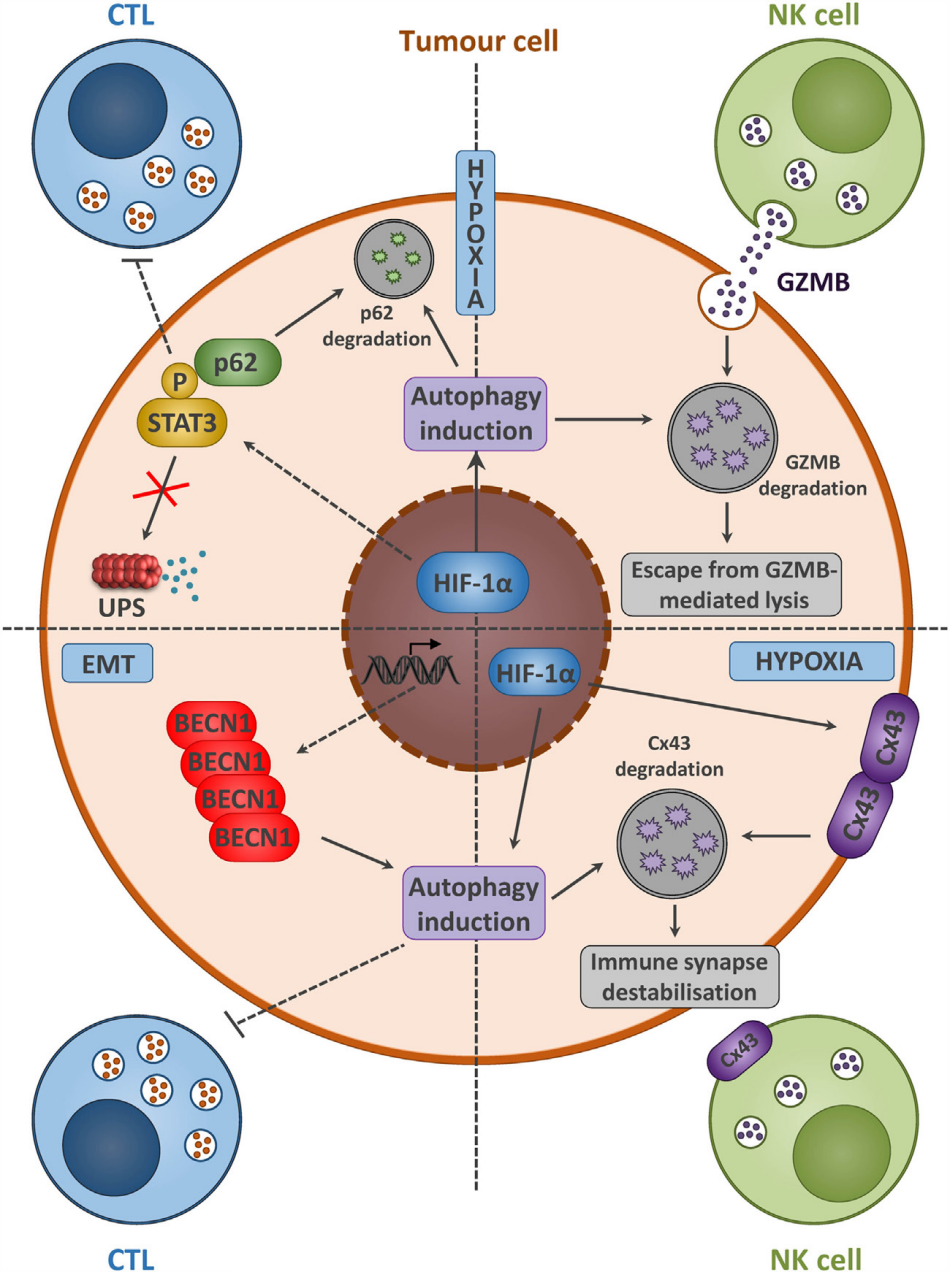
**Tumor cells with activated autophagy defend themselves from NK- and CTL-mediated killing by diverse mechanisms**. Hypoxia and/or EMT activation induces autophagy in tumor cells. A summary of diverse mechanisms by which autophagy activation leads to the acquisition of tumor cell resistance to CTL and NK-mediated lysis. Hypoxia-induced autophagy in cancer cells acts as an intrinsic resistance mechanism to NK-mediated lysis through the selective degradation of the NK-derived serine protease granzyme B (GZMB) (upper right). Moreover, the selective degradation of the gap junction protein connexin 43 (Cx43) is also implicated in the destabilization of the interaction between tumor and NK cells (lower right). The activation of the signal transducer and activator of transcription 3 (STAT3) signaling along with autophagy activation in tumor cells leads to the acquisition of resistance to CTL-mediated lysis (upper left). Autophagy activation, following epithelial-to-mesenchymal (EMT) induction, leads to BECN1 overexpression, and is sufficient to overcome CTL-mediated attack (lower left).

It is now clearly established that autophagy activation in cancer cells is an effective way to communicate with the tumor microenvironment. However, accumulating observations have highlighted that autophagy activation may also serve as an intrinsic mechanism of resistance evolved by cancer cells to overcome immune cell attack (Figure 3) (13). Noman et al. demonstrated that hypoxia-induced autophagy acts as a mechanism of resistance to cytotoxic T cells-mediated lysis through the activation of the signal transducer and activator of transcription 3 (STAT3) signaling (50, 51). Our group provided for the first time direct evidence between autophagy and the regulation of STAT3 signaling. Inactivation of autophagy, by specific silencing of BECN1 or ATG5, restored lung cancer cell sensitivity to T cells lysis, which was associated with a decrease in hypoxia-induced pSTAT3. Interestingly, *in vivo* administration of the autophagy inhibitor hydroxychloroquine (HCQ) with a tyrosinase-related protein-2 (TRP2) peptide-based vaccination strategy led to a significant reduction of melanoma growth compared to vaccine or HCQ treatment alone. Although the clear link between autophagy activation and induction of STAT3 signaling remains to be elucidated, it has been clearly established that STAT3 is an important mediator in the crosstalk between tumor and immune cells (Figure 3) (52). The aberrant STAT3 signaling in tumor cells can suppress the expression of pro-inflammatory danger signals (e.g., IFNγ, TNF, CXCL10), while induces the expression of immunosuppressive factors (e.g., VEGF, IL-10) that inhibit DCs maturation (53).

More recently, studies have reported that tumor cells undergoing epithelial-to-mesenchymal transition (EMT) escape from T cell-mediated lysis. During EMT, phenotypic changes occur and expression of certain tumor antigens is downregulated, thus restraining T cells specific recognition and killing (54). The consequence of EMT on immune system is likely to be dependent on the cancer cell origin. Indeed, depending on the tumor type, EMT may either avoid proliferation/stimulate apoptosis of NK, B, and T cells or promote expansion of Tregs (55). The relationship between autophagy and EMT is not clearly established so far. However, it has been reported that acquisition of mesenchymal phenotype in breast cancer cell was associated with modification of autophagy gene expression, indicating a concomitant autophagy activation in those cells (56). EMT induction through the overexpression of snail homolog 1 (SNAI1) in MCF7 breast cancer cell line led to autophagy induction through the upregulation of BECN1. These observations indicated that autophagy activation is a downstream target of EMT. Thus, targeting EMT-induced autophagy in mesenchymal cells was sufficient to restore T cell-mediated lysis without affecting cell morphology and the expression of EMT markers (Figure 3) (57, 58).

#### Autophagy Modulates the Innate Antitumor Immune Response

Our group also provided evidence that autophagy induction in hypoxic breast cancer cells influences the innate antitumor immunity mediated by NK cells (59, 60). We demonstrated *in vitro* that the NK-derived serine protease granzyme B (GZMB) was selectively degraded into autophagosomes in hypoxic breast cancer cells, in which autophagy is induced. Selective inhibition of autophagy, by silencing BECN1 or ATG5 in hypoxic cancer cells, restored the susceptibility to NK-mediated lysis. This concept was validated *in vivo* by using two syngeneic models of tumor transplantation. We showed that inhibition of autophagy in cancer cells contributed to a significant reduction of the tumor volume by improving elimination by NK cells (59). In line with this study, the role of autophagy in modulating the NK-mediated antitumor immune response was extended to ccRCC. The VHL-mutated ccRCC, exhibiting high autophagy rate, was less susceptible to NK-derived GZMB than VHL-corrected ccRCC. This resistance required the stabilization of HIF-2α and the subsequent overexpression of the autophagy sensor inositol 1, 4, 5-trisphosphate receptor, type 1 (ITPR1). Both inhibition of BECN1 or ITPR1 in VHL-mutated ccRCC cells restored NK-mediated killing (Figure 3) (61, 62).

Additionally, activation of autophagy in cancer cells was shown to be implicated in the destabilization of the interaction between target and effector cells. Hypoxia-induced autophagy was identified as a selective degradation pathway of the gap junction protein connexin 43 (Cx43) that renders the melanoma cells resistant to NK cells-mediated attack. Expression of a non-degradable form of Cx43 or targeting autophagy in hypoxic cancer cells restored the accumulation of Cx43 at the immunological synapse and improved NK cells-mediated lysis (Figure 3) (63).

Finally, autophagy induction is known to be closely related to the pathway of exosome secretion (64). In addition, hypoxia was described to enhance exosome release by cancer cells (65). Exosomes are small extracellular vesicles of endosomal origin, are released by all cell types, and constitute a new component of cell–cell communication. They carry membrane proteins, enzymes, chaperone proteins, but also DNA and various types of RNA (mRNA, lncRNA, and miRNA). Microvesicles derived from hypoxic cells were shown to induce an invasive and pro-metastatic phenotype (66, 67) and to inhibit the immune response (68). In particular, we showed that vesicles produced by hypoxic cancer cells inhibit NK cell functions. Following their uptake, the vesicles transfer TGF-β1 to NK cells, decreasing expression of the activating receptor NKG2D on the cell surface. We also identified high levels of miR-23a in hypoxic vesicles that directly targets the expression of the marker of degranulation CD107a in NK cells. Those two immunosuppressive mechanisms inhibiting NK cell functions strongly contribute to decrease the antitumor immune response (68).

## Autophagy Inhibition Along with New Combinatorial Immune Checkpoint Blockers

Immune checkpoint-based immunotherapy revolution has just started, and new combination strategies with potent curative potential are currently emerging (22). In spite of outstanding and encouraging response, the majority of patients treated with anti-PD-1/PD-L1 monotherapies have partial tumor regressions and they fail to achieve higher objective responses. This suggests that in order to obtain durable tumor regressions, there is a need for combination therapies along with anti-PD-1/PD-L1 monotherapies. Several reports using various preclinical animal models have shown that combination therapies based on immune checkpoint blockers are strongly synergistic along with classical cancer therapies (including chemotherapy, tyrosine kinase inhibitors, radiotherapy, and vaccines) (21).

The combination of both anti-CTLA-4 and anti-PD1 has been shown to be twice as effective as either treatment alone in the rejection of B16-F10 melanoma tumors. This combination therapy increased T-effector to MDSC ratio and also increased IFN-γ/TNF-α double producing CD8+ T cells. Finally, PD-1 and CTLA-4 combination blockade expanded infiltrating T cells and reduced regulatory T and myeloid cells within B16 melanoma tumors (69). Similarly, in mouse models of colon carcinoma (CT26 cell line) and ovarian carcinoma (ID8-VEGF cell line), dual block (both anti-PD1 and anti-CTLA-4) combined with tumor vaccine leads to effective tumor rejection through the restoration of effector T cell function (70). More interestingly, it has been reported that the combination of anti-4-1BB and anti-PD-1 is more effective than that of anti-PD-1/anti-LAG-3 in suppressing B16-F10 melanoma tumor growth without adjuvant or vaccination (71). In a phase 1 clinical trial with advanced melanoma patients, the combination of anti-CTLA-4 and anti-PD-1 increased the response rate up to 53% patients with severe treatment-related adverse events (72). Autophagy inhibitor CQ is currently used in several clinical trials to sensitize tumor cells to radio and chemotherapy. We have previously shown that, by simultaneously boosting the immune system and inhibiting autophagy using CQ, we can significantly enhance the therapeutic efficacy of antigen-based cancer vaccines in B16 tumors (51). In this regard, a combination of autophagy inhibition and immune checkpoint-based immunotherapy would probably revert immune suppressive microenvironment and promote additional tumor regression. Although anti-CTLA-4 and anti-PD-1 are currently the focus of clinical attention, it is likely that blockade of additional checkpoints will result in even further clinical activity as multiple checkpoints appear to be co-expressed with PD-L1 and PD-1 on the same tumors (21, 22).

## Concluding Remarks

Cancer immunotherapy based on immune checkpoint inhibitors marks a turning point in cancer treatment since it revolutionizing the way we treat cancer. Immune checkpoint-based immunotherapies have shown great promise for a subset of cancer patients. However, safe, robust, and attentive combination therapies are still needed to bring the benefit of cancer immunotherapy to a larger class of cancer patients. While searching for an optimal strategy of combinatorial immunotherapy, we have to keep in mind that different immune checkpoints are expressed simultaneously on both tumor cells, stromal cells, and different immune cells. Furthermore, there is a differential expression of both inhibitory and activating receptors on different immune cells within the tumor microenvironment. Autophagy remains to be a pretty interesting combination target while developing novel and cutting edge cancer immunotherapeutic approaches.

## Author Contributions

EV, MN, TA, AL, SC, GB, EM, JP, and BJ contributed to the writing of the manuscript and the conception of the figures.

## Conflict of Interest Statement

The authors declare that the research was conducted in the absence of any commercial or financial relationships that could be construed as a potential conflict of interest.
